# The Negligible Influence of Chronic Obesity on Hospitalization, Clinical Status, and Complications in Elective Posterior Lumbar Interbody Fusion 

**DOI:** 10.1155/2016/2964625

**Published:** 2016-07-10

**Authors:** Olaf Suess, Theodoros Kombos, Frank Bode

**Affiliations:** ^1^Zentrum fuer Wirbelsaeulenchirurgie und Neurotraumatologie, DRK Kliniken Berlin Westend, Spandauer Damm 130, 14050 Berlin, Germany; ^2^Neurochirurgische Klinik, Schlosspark Klinik, Heubnerweg 2, 14059 Berlin, Germany; ^3^Neurochirurgische Klinik, Klinikum Darmstadt, Grafenstrasse 9, 64283 Darmstadt, Germany

## Abstract

*Background*. Posterior lumbar interbody fusion (PLIF) is a common surgical treatment for degenerative spinal instability, but many surgeons consider obesity a contraindication for elective spinal fusion. The aim of this study was to analyze whether obesity has any influence on hospitalization parameters, change in clinical status, or complications.* Methods*. In this prospective study, regression analysis was used to analyze the influence of the body mass index (BMI) on operating time, postoperative care, hospitalization time, type of postdischarge care, change in paresis or sensory deficits, pain level, wound complications, cerebrospinal fluid leakage, and implant complications.* Results*. Operating time increased only 2.5 minutes for each increase of BMI by 1. The probability of having a wound complication increased statistically with rising BMI. Nonetheless, BMI accounted for very little of the variation in the data, meaning that other factors or random chances play a much larger role.* Conclusions*. Obesity has to be considered a risk factor for wound complications in patients undergoing elective PLIF for degenerative instability. However, BMI showed no significant influence on other kinds of peri- or postoperative complications, nor clinical outcomes. So obesity cannot be considered a contraindication for elective PLIF.

## 1. Introduction

Degeneration of the spine is a leading cause of pain and disability [1–3]. This degeneration alters the discoligamentary structures and often leads to spinal instability. Surgical fusion of the affected spinal segments has long been established as the best available surgical treatment for such instability. Posterior lumbar interbody fusion (PLIF) specifically has been the most common approach to fusion [4, 5].

Obesity is thought to represent a risk factor, or even a contraindication, for spinal surgery, as obese patients often have significant comorbidities. Preoperative imaging may be of lower quality. Anesthesia management may be more difficult. The surgical approach to the bony structures of the spine is made more difficult by the excessive subcutaneous fat deposits. That is why surgeons often have to increase the incision length to allow adequate visualization at the increased depth for obese patients, and it is thought that this may lead to increased complications, especially wound infection.

Several previous studies have reported on the influence of obesity on outcomes and complications for spinal surgery. Most notably, in a prospective study of 150 patients undergoing lumbar spine surgery with one-year follow-up, Andreshak et al. found no significant differences between obese and control patients and concluded that lumbar spine surgery should not be withheld from obese patients who are otherwise appropriate candidates [6]. In a retrospective study of 850 spinal procedures in 521 patients, Wimmer et al. found obesity to be a risk factor for infection, but methodological details on this point were sparse [7]. Similarly, in a retrospective case-control study of 41 cases and 178 controls undergoing laminectomy and/or fusion, Olsen et al. identified morbid obesity (BMI > 35) as a risk factor for surgical site infection and recommended that they receive higher doses of prophylactic antibiotic agents and insulin pumps to maintain serum glucose levels below 200 mg/dL [8]. In analysis of data from a multicenter, prospective, randomized controlled trial involving 75 circumferential fusion patients, Goldstein found that BMI had no effect on functional improvement, pain improvement, or patient satisfaction [9]. In a retrospective study of 84 patients receiving fusion for lumbar or thoracic degeneration, Patel et al. did find that obesity was significantly related to major complications but not to minor ones [10]. In a retrospective review of 497 patients undergoing fusion, Glassman et al. noted that BMI had no influence on physical quality-of-life (SF-36 PCS) or functionality (ODI) at 1 or 2 years post-op [11]. In a retrospective chart review of 43 ICLF patients receiving Worker's Comp, LaCaille et al. found that obesity was significantly related to total worker's compensation received but not to total medical costs [12]. Thus although there is some evidence that obesity is a risk factor for wound infection [7, 8] and other complications [10], the literature mostly shows that obesity does not negatively influence outcomes [6, 9, 11] and usually concurs that obesity is not a contraindication for PLIF [6, 8, 9, 11].

In clinical practice though obesity is often still regarded as a contraindication for elective spinal instrumentation. This may represent more an unacknowledged bias against overweight people rather than best-evidence medicine, though the scientific literature is not yet entirely consistent and conclusive. Considering that obesity is already pandemic in Europe and growing worldwide [13–15], it is crucial to scientifically determine whether or not it really is a contraindication against surgery. So we undertook an analysis of 78 patients who had undergone PLIF at our department over a two-year period. The aim of this study was to search for any evidence that obesity has a negative influence on hospitalization, clinical status, or complications.

## 2. Methods

### 2.1. Patients and Surgery

The research was designed as a prospective cohort study. All patients presenting to our department between 06/2006 and 06/2007 were candidates for this study. The inclusion criteria were (1) mono- or bisegmental lumbar instability, confirmed clinically and radiologically and (2) persistence of symptoms despite conservative treatment. The following radiological measurements have been used to establish lumbar instability. Direct signs of instability are as follows: 3 mm or more of anterior translation measured between flexion and extension X-ray and/or abnormal axial rotation in which posterior margin of the vertebral body has a focal double contour during bending. Indirect signs of instability are as follows: disc degeneration with space narrowing, osteosclerosis, and osteophytosis of the vertebral end plates. Furthermore, the presence of traction spur, which is a particular type of osteophyte that is located 2-3 mm from the end plate and has a horizontal orientation, and presence of an intervertebral vacuum phenomenon were considered as indirect signs of instability.

As a clinical sign, aberrant motions such as the instability catch occurring during active ROM testing was used to indicate instability. The instability catch has been described as a sudden acceleration or deceleration of movement or a movement occurring outside of the primary plane of motion (e.g., side bending or rotation occurring during flexion) and is proposed as an indication of segmental lumbar instability. Other clinical tests for the evaluation of instability included Gower's sign (thigh climbing to return from a flexed to an upright position), hypermobility during spring testing (posterior-to-anterior (PA) glide), pain during spring testing, and increased muscle guarding or muscle spasm. The exclusion criteria were (1) previous spinal surgery, (2) improvement of symptoms through conservative treatment, and (3) uncorrelated patient symptom complaints. Underweight patients (BMI < 18.5) were excluded from the present analysis.

The surgery followed standard procedures for PLIF, as described in the literature. In brief, mono- or bisegmental laminectomy was performed (according to the number of levels to be fused). Dural sac and nerve roots were relieved from discoligamentary compression. After localization of the pedicles of the vertebrae to be fused, polyaxial 6.5 mm pedicle screws (XIA Low Profile or XIA Precision, Stryker, USA) were introduced with discontinuous fluoroscopy. The intervertebral space was cleared, olisthesis was corrected by retraction if necessary, and finally bilateral intervertebral PEEK cages (Wave, AMT, Germany) were introduced and filled with autologous bone graft. The pedicle screws were blocked and fixed with lateral rods under mild compression. Finally, the wound was closed in typical manner with multilayer resorbable sutures. Antibiotics (2nd generation cephalosporins) were given as a single shot prophylaxis 20 minutes prior to skin incision.

### 2.2. Measurements

Standardized questionnaires (including ODI and SF36) were used to document sociodemographic variables and preoperative risk factors. BMI was determined by physical examination and graded as normal ≥ 18.5–25; overweight ≥ 25–30; obese ≥ 30–35, severely obese ≥ 35–40, and morbidly obese ≥ 40. Preoperative spondylolisthesis was assessed radiographically and scored by Meyerding's grade. Operating time and hospitalization duration were recorded by hospital staff. Immediate post-op care (defined as the first 12 hours after surgery) was recorded as neurosurgical ward, intermediate monitoring unit, or intensive care unit (ICU). Postdischarge care was recorded as home/self, outpatient treatment, or rehabilitation clinic. Motor strength was measured on a BMRC grading scale (0/5–5/5), for L3-S1, preoperatively and postoperatively. Dermatome-specific sensory deficits were recorded as absent or present left and right, for L3-S1, preoperatively and postoperatively. Adductor reflexes, patellar tendon reflexes, and Achilles' tendon reflexes were each tested left and right and scored on a scale of 0–3, preoperatively and postoperatively. Urogenital disorders, spinal claudication, and Lasègue's sign were each assessed clinically as present versus absent, preoperatively and postoperatively. Pain symptoms were quantified preoperatively and postoperatively using a 10-point visual analogue scale (VAS) and recorded semiquantitatively as “none” (0), “light” (1–3), “moderate” (4–6), or “severe” (7–10). Complications were recorded individually in the patient records.

### 2.3. Statistical Analysis

Statistical analysis was performed to test the influence of BMI on hospitalization parameters, clinical outcomes, and complications. In all analyses, the following factors were controlled for sex, age, Meyerding grade, and preoperative risk factors. Hospitalization parameters included operating time, type of immediate post-op care, hospitalization duration, and discharge facility. Multiple linear regression was used to analyze operating time, controlling additionally for the number of spinal levels being fused, and the presence of osteoporosis. Multinomial regression was used to analyze the influence of BMI on post-op care. Cox proportional hazard regression was used for hospitalization duration. Multinomial regression was used to analyze the influence of BMI on postdischarge care.

Clinical outcomes included change in paresis, change in sensory deficits, change in reflexes, and change in pain level. For statistical analysis, the motor strength scores for all levels left and right were summed for each pre-op and post-op, and the change in paresis was calculated as the post-op sum minus the pre-op sum; an identical procedure was used for sensory deficits and to sum the three reflex measurements, left and right. The influence of BMI on each paresis, sensory deficits, and reflexes was analyzed first with multiple regression treating the outcome measures as continuous variables, and then alternatively with logistic regression comparing improvement (post-op score − pre-op score = 1 or greater) to no improvement or deterioration (post-op − pre-op = 0 or less). Logistic regression was used to assess the influence of BMI on change in bladder/colon dysfunction, spinal claudication, and Lasègue's sign, individually. Ordinal regression was used to analyze the influence of BMI on change in pain level.

Complications included wound, implant, and cerebrospinal (CSF) complications. “Wound complications” was the sum of wound healing disorders, wound infections, and surgical revision of wounds; due to the small number of cases with two or more wound complications, this variable was dichotomized as 0 versus 1 or more. Logistic regression was used, controlling here additionally for operating time. “Implant complications” was the sum of device loosening and device breakage; again, this variable was dichotomized as 0 versus 1 or more, and logistic regression was used. “Cerebrospinal (CSF) complications” was the sum of dura injuries, nerve root injuries, cerebrospinal fluid fistulas, and cerebrospinal fluid accumulation; again, this variable was dichotomized as 0 versus 1 or more, and logistic regression was used.

The data met all the necessary assumptions for use of these regression models. Statistical analysis was performed with Stata 10.0 (Stata, College Station, Texas, USA). Statistical graphing was with performed with Stata 10.0 (Stata, College Station, Texas, USA) and Adobe Illustrator 10 (Adobe; San Jose, CA, USA).

## 3. Results

### 3.1. Patient Population

**Figure 1:**
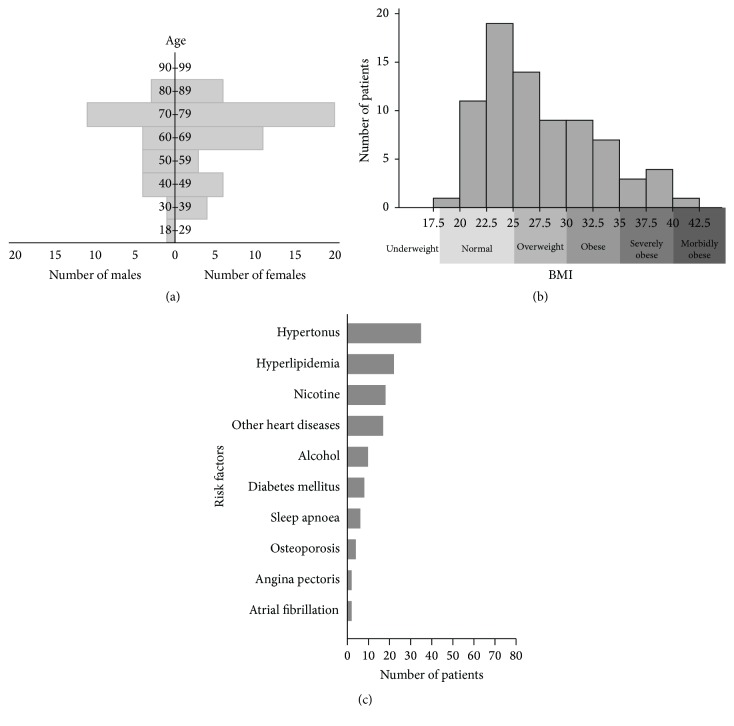
Patient characteristics. (a) Population pyramid, showing the sex and age distribution of the study sample. (b) Histogram of BMI. (c) Bar graph of the incidence of risk factors (see Table 1).

**Figure 2:**
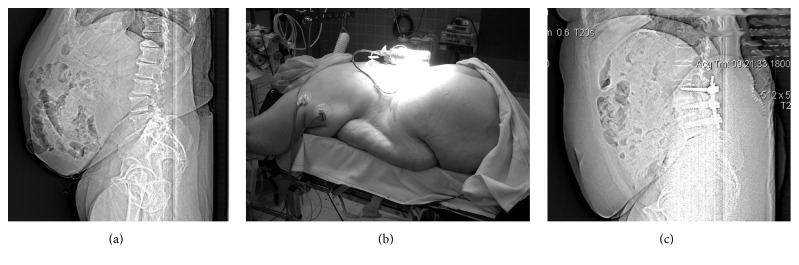
Example of obese patients with BMI > 30. (a) Presurgical CT scout of a 64 y.o. male patient with BMI 34.6. (b) Severely obese 59 y.o. female patient with BMI 37.1 positioned on the OR-table. (c) CT scout of the patient shown in (c) after two-level PLIF.

The study included 78 patients. The patients' sex and age are shown in Figure 1(a). The distribution of the patients' BMIs is shown in Figure 1(b). The distribution of patients according to spinal level(s) in need of operation and grade of preoperative spondylolisthesis is shown in Table 1. The incidence of risk factors in this patient cohort is shown in Figure 1(c). Twenty-four of the 78 patients (30.8%) had a BMI > 30 and were regarded as being obese (16/24), severely obese (7/24), or morbidly obese (1/24) (for examples, see Figures 2(a)–2(c)).

### 3.2. Hospitalization Parameters

**Figure 3:**
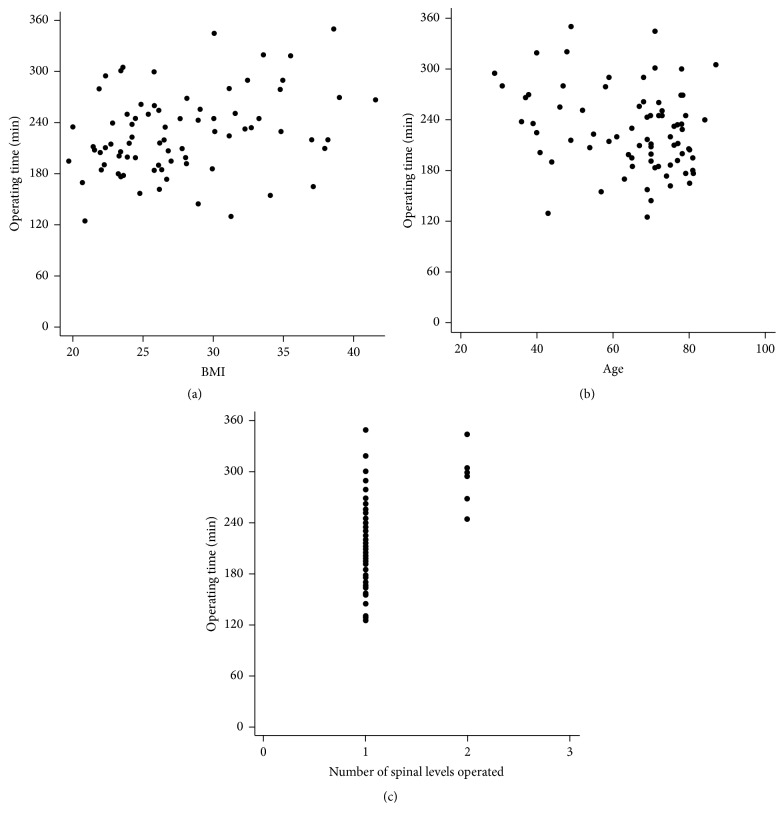
Influence of BMI on operating time. The graph shows a scatter-plot of the BMI and operating time data. The thumbnails are similar scatter-plots showing the influence on operating time from age and numbers of levels fused, which were also significant in the overall regression model. It should be kept in mind that each graph shows only the bivariate distribution of operating time and one other variable; the graphs cannot visually adjust or control for the other two significant variables. The statistical parameters for this regression model were as follows: regression slope = 2.56, 95% CI = 0.79 to 4.35, *t* = 2.87, *p* = 0.005, and adjusted *r* ^2^ = 0.31.

The median (range) operating time was 220 (125–350) minutes (Table 2). BMI had a statistically significant influence on operating time, when controlling for age and number of levels operated (Figure 3). Operating time increased by 2.5 minutes for each BMI increase of 1, after controlling for age and levels operated, thus, for example, predicting that operating on a patient with a BMI of 40 would take 50 minutes longer than on a patient with a BMI of 20.

Immediate post-op care was on the neurosurgical ward for 73 (93.6%) patients, whereas 5 patients (6.4%) needed an intermediate monitoring unit for up to 48 hours post-op due to cardiopulmonary complications; no patients were on ICU. BMI had no significant influence on the patients' immediate post-op care. The median (interquartile range and min–max range) hospitalization duration was 15 (12–20; 7–92) days. BMI had no significant influence on the duration of hospitalization. The postdischarge care was home/self for 38 (48.7%) patients, outpatient care for 36 (46.2%) patients, and inpatient rehabilitation for 4 (5.1%) patients. BMI had no significant influence on the patients' type of postdischarge care.

### 3.3. Clinical Status

The median (interquartile range) change of the sum scores for paresis 0 (0-0), sensory deficits 0 (0-1), and reflex status 0 (0-0) showed that BMI had no significant influence on motor or sensory outcome. Furthermore, BMI had no significant influence on the pre-op to post-op change of urogenital dysfunction, spinal claudication, or Lasègue's sign. The median (interquartile range) change of pain level was 2 (2-3) levels. BMI had no significant influence on change in pain level.

### 3.4. Complications

**Figure 4:**
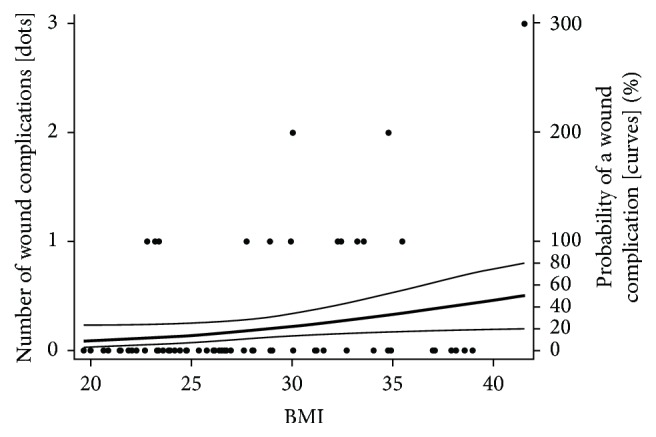
Influence of BMI on wound complications. The graph shows a scatter-plot of BMI and the number of wound complications, with the regression curve and 95% CI superimposed using the right-side axis. Dots represent the actual data of BMI and wound complications from this study. The middle curve represents the probability of a wound complication, and the upper and lower curves show the interval within which the probability curve can be assumed with 95% confidence to lie. The statistical parameters for this model were as follows: regression slope = 0.11, 95% CI = 0.005 to 0.217, *z* = 2.28, *p* = 0.04, and pseudo *r* ^2^ = 0.07.

There was delayed wound healing in 12 (15%) patients, a bacterial wound infection in 2 (2.6%) patients and a need for surgical wound revision (due to CSF leakage or deep wound infection) in 5 (6.4%) patients; when dichotomized, 15 (19.2%) patients had one or more wound complications. BMI had a statistically significant influence on wound complications (Figure 4). In addition to being statistically significant, the actual magnitude of the influence of BMI on the risk for a wound complication was clinically substantial (as can be seen by the large rise in the probability curve in Figure 4). Thus for illustration, the probability of having a wound complication rose from about 8% at a BMI of 20 to about 33% at a BMI of 35. Despite being statistically significant and clinically substantial, BMI had weak explanatory power for the actual occurrence of a wound complication (as can be seen by the fact that the data dots in Figure 4 cluster only weakly as one looks from the bottom left to the top right and by the wideness of the 95% CI above and below the probability curve in Figure 4). BMI accounted for only about 5% of the variance in the data and therefore cannot be regarded as more than a minor factor in explaining which patients actually have a wound complication. Yet no other control variable in our analysis, including operating time, had a significant influence on wound complications. This implies that random chance or other uncaptured factors account for the other 95% of variance in the data.

There were 1 (1.3%) instance of device breakage and 3 (3.8%) cases with radiological signs of nonfusion and device loosening after 12-month follow-up. BMI had no significant influence on the occurrence of an implant complication. There were 6 (7.7%) cases of intraoperative dura injuries and 4 (5.2%) cases of postoperative cerebrospinal fluid fistulas. BMI had no significant influence on the occurrence of a cerebrospinal complication.

## 4. Discussion

In this study, we found an influence of BMI only on operating time and wound complications. BMI had no significant influence on any of the other hospitalization parameters, clinical outcomes, or complications analyzed here. Although the operating time increased with rising BMI, the magnitude of additional operating time was small and all patients were still within the range of typical operating time for spinal surgery. Moreover, operating time has not been proven to have any convincing clinical relevance. BMI did substantially increase the probability of having a wound complication. But a higher risk for a wound complication cannot be viewed as sufficient grounds to contraindicate back pain patients for PLIF. As Olsen et al. have explained in greater detail [8], the appropriate response to this finding is simply that surgeons need to take more preventive measures when operating on obese patients, including, for example, doubling the dose of antibiotics used and using an insulin pump to maintain serum glucose levels under 200 mg/dL. Furthermore, obese patients should simply be informed that they have a higher risk of wound complication, so extra preventive measures will be taken if they decide to opt for the surgery anyway [16–18]. It should also be emphasized that BMI explained very little of the variation in the data, meaning that other factors are the main explanations of which patients actually have a wound complication. So if our concern is to avoid wound complications, further research is needed on the factors that lead to them [19]. Besides operating time and wound complications, BMI had no significant influence on any of the variables analyzed here.

No one study can single-handedly settle any scientific questions, including whether or not obesity is a contraindication for spinal surgery. Only the overall body of literature can answer the question. The present findings are consistent with most other studies. The only anomalous exception has been the study by Patel et al. [10], which had a study size, sample, and design comparable to our own. They had a much lower rate of wound infection (3 cases in 84 patients versus our 15 cases in 78 patients), but a higher rate of cerebrospinal fluid leakage (8 cases versus our 4 cases of cerebrospinal fluid fistulas or accumulation). They also reported several complications that we never observed, including deep vein thrombosis, cardiac events, pneumonia, prolonged intubation, urological issues, and palsy. The occurrence of all these other complications cannot be due solely to obesity; otherwise, we (and other researchers) would have observed at least some of them too. Instead, these complications are probably due to general health differences in these (American versus German) study populations or differences in the hospital setting. Furthermore, the rate of complications observed by Patel et al. appears to be rather high in comparison to most other previous studies on spine surgery in obese patients. This might be due in part to the broad definition of “complications” that they used, yet it remains difficult to understand why they had so many major complications such as cardiac events and pneumonia. Several other studies have reported no difference in clinical outcomes for obese patients and no such long lists of major complications [6, 8, 9, 11, 12].

## 5. Conclusions

Obesity is well-known to be a risk factor for heart disease, diabetes, and other medical conditions, and it is associated with higher rates of health care consumption [15, 20–22]. However, according to the results of this study and consistent with the bulk of currently available evidence, obesity is not clearly associated with enough increased risk of complications or poor outcomes to be viewed as a contraindication for PLIF.

## Figures and Tables

**Table 1:** Box chart of spinal levels operated and Meyerding grade.

		Meyerding grade				
		1	2	3	4	Total
Spinal level(s) operated	L2/L3	2	1	0	0	**3**
	L3/L4	4	3	0	0	**7**
	L4/L5	38	11	1	0	**50**
	L5/S1	6	5	1	0	**12**
	L3/L4 + L4/L5	3	0	0	0	**3**
	L4/L5 + L5/S1	2	1	0	0	**3**
	Total	**55**	**21**	**2**	**0**	**78**

**Table 2:** Summary of influence of BMI on hospitalization parameters, clinical status, and adverse events. Multinomial regression was used to analyze the influence of BMI (body mass index) on post-op care, Cox proportional hazard regression was used for hospitalization duration, and multinomial regression was used to analyze the influence of BMI on postdischarge care. The influence of BMI on motor and sensory function was analyzed with multiple regression first, as well as logistic regression comparing postsurgical improvement scores. Logistic regression was used to assess the influence of BMI on change in urogenital dysfunction and spinal claudication as well as on the appearance of adverse events. Ordinal regression was used to analyze the influence of BMI on change in pain level.

		Influence of BMI
*Hospitalization parameters*		
Operating time	220 min (125–350) mean (min–max range)	*Moderate influence*: (+2.5 min for each BMI increase of 1)
Immediate post-op care		No influence
Neurosurgical ward	73 (93.6%)	
Monitoring unit	5 (6.4%)	
Intensive care unit	0 (0%)	
Hospitalization duration	15 days (12–20; 7–92) mean (interquartile range, min–max range)	No influence
Post discharge care		No influence
Home	38 (48.7%)	
Outpatient care	36 (46.2%)	
Inpatient rehabilitation	4 (5.1%)	

*Postsurgical clinical status*		
Motor function		No influence
Sensory function		No influence
Urogenital function		No influence
Claudication		No influence
Pain level (VAS)		No influence

*Complications*		
Wound healing problems	12 (15.4%)	*Minor influence*: (Probability 8% at BMI 20, 33% at BMI 35; BMI accounts for only 5% of variance)
Bacterial wound infections	2 (2.6%)	
Surgical wound revisions	5 (6.4%)	
Screw/implant breakage	1 (1.3%)	No influence
Implant loosening/nonfusion	3 (3.8%)	No influence
Dura injuries	6 (7.7%)	No influence
Cerebrospinal fluid fistula	4 (5.1%)	No influence
